# Pan-Cancer, Genome-Scale Metabolic Network Analysis of over 10,000 Patients Elucidates Relationship between Metabolism and Survival

**DOI:** 10.3390/cancers16132302

**Published:** 2024-06-22

**Authors:** Jesse Bucksot, Katherine Ritchie, Matthew Biancalana, John A. Cole, Daniel Cook

**Affiliations:** SimBioSys, Inc., 180 N La Salle St., Chicago, IL 60601, USA

**Keywords:** systems biology, pan-cancer, metabolic modeling, prognosis, therapy repurposing

## Abstract

**Simple Summary:**

This study describes a large-scale analysis of cancer metabolism across The Cancer Genome Atlas and Multiple Myeloma Research Foundation MMRF-COMMPASS study encompassing 10,915 patients from 34 different cancer types. To the best of our knowledge, this is the first such large-scale metabolic analysis performed using these data. Our approach identified biologically interpretable metabolic biomarkers of overall survival in 31 out of 34 cancer types evaluated. Additionally, in a subset of 7 cancer types, we predict chemosensitivity to antifolate-based therapies and a biomarker of response based on our patient-specific metabolic models. Finally, this work introduces the concept of “interpretation-driven biomarker identification”. In this framework, biological interpretability of biomarker signatures is ensured first by using network-based simulations to characterize tumor biology, then using survival analysis to identify any biological features associated with overall survival—essentially decoupling biomarker discovery and biological interpretation of biomarker scores.

**Abstract:**

Despite the high variability in cancer biology, cancers nevertheless exhibit cohesive hallmarks across multiple cancer types, notably dysregulated metabolism. Metabolism plays a central role in cancer biology, and shifts in metabolic pathways have been linked to tumor aggressiveness and likelihood of response to therapy. We therefore sought to interrogate metabolism across cancer types and understand how intrinsic modes of metabolism vary within and across indications and how they relate to patient prognosis. We used context specific genome-scale metabolic modeling to simulate metabolism across 10,915 patients from 34 cancer types from The Cancer Genome Atlas and the MMRF-COMMPASS study. We found that cancer metabolism clustered into modes characterized by differential glycolysis, oxidative phosphorylation, and growth rate. We also found that the simulated activities of metabolic pathways are intrinsically prognostic across cancer types, especially tumor growth rate, fatty acid biosynthesis, folate metabolism, oxidative phosphorylation, steroid metabolism, and glutathione metabolism. This work shows the prognostic power of individual patient metabolic modeling across multiple cancer types. Additionally, it shows that analyzing large-scale models of cancer metabolism with survival information provides unique insights into underlying relationships across cancer types and suggests how therapies designed for one cancer type may be repurposed for use in others.

## 1. Introduction

Cancer is a highly heterogeneous disease, varying both across patients and within single tumors. This heterogeneity manifests across numerous cellular features, including variability on the genomic scale (with clonal expansions and evolutionary pressures), on the transcriptomic scale (with gene fusions and altered transcriptional regulation), on the protein scale (with altered expression and activity profiles), on the metabolic scale (with altered mitochondrial function and glycolysis utilization), and even on the tumor microenvironment scale (with differences in infiltration of lymphocytes into the tumor, activation of cancer associated fibroblasts, and altered balances among different types of T cells). This incredible diversity of behavior demands a rigorous, integrative methodology for assessing the underlying mechanism(s) supporting cancer proliferation and response to therapy [1].

Despite the unique profile of individual tumors as well as intra-tumoral variability, certain themes emerge time and again in cancer biology. The first identified of these was the Warbug effect, discovered at the turn of the century, which characterized cancer’s propensity to increase glycolysis in relation to oxidative phosphorylation compared to non-cancerous tissue. Since then, 23 “hallmarks of cancer” have been identified [2,3,4], with further mechanistic features continuing to be proposed [5]. Such instances of convergent evolution suggest that similar biological features may govern cancer aggressiveness, intrinsic susceptibility to specific therapies, and development of resistance.

Recent research has revealed how these commonalities across cancer types and between patients are associated with target identification, therapy selection, and overall patient prognosis. Specific gene signatures have been identified across an array of cancers that distinguish malignant and normal tissue [6]. Intriguingly, signatures that are able to predict patient survival in one cancer type have been shown to translate to other forms of cancer, suggesting a shared set of genetic properties that may underlie the chances of response to therapy, recurrence, or both [7]. Additionally, statistical techniques have been used to propose prognostic gene expression signatures across multiple cancer types [8,9]. Defining the signatures underpinning these commonalities between cancer types, however, has been plagued by challenges, including overfitting to limited training data, variability in data quality, lack of mechanistic insight from signatures, and lack of reproducibility [10,11,12]. Network-based analyses have therefore emerged as a mechanism to account for these challenges. For instance, a network-based algorithm analyzing data from The Cancer Genome Atlas (TCGA) and the human protein-protein interaction (PPI) network was employed to identify a 41 gene signature that stratified good vs. poor prognosis in 8 out of the 11 cancers used to train the algorithm, which was prognostic in independent datasets [13]. Similar network-based analyses performed on a per-cancer-type basis have also suggested biologically interpretable prognostic gene signatures for specific cancer types [14].

The thought process underlying the development of these techniques reveals a common perspective among current approaches to biomarker discovery. That perspective states that predictive biomarkers should be based on the prognostic power of individual genes (or sets of genes), and the biological interpretation of these gene sets can be extracted after biomarker identification—either through gene set enrichment or network analysis. This approach can be summarized as: gene set first, interpretation after. Our approach, in contrast, represents a different perspective: we call it interpretation-driven biomarker identification. We first use network-based simulations to characterize the biology at work across cancers; we then use survival analyses to identify any biological features associated with overall survival within and across cancer types. Our approach prioritizes biological interpretability of the resulting features by pre-selecting which gene sets (or pathways) will be included in each feature and their weights, prior to testing these sets against survival data. The specific type of network-based simulations used in this work were context-specific Flux Balance Analysis (FBA) on a human metabolic reconstruction to understand metabolism across multiple tumor types from TGCA and the MMRF-COMMPASS study. Metabolism is an excellent test case for such an analysis because of its importance for tumor biology and for influencing growth rate, as well as the maturity of tools for simulating metabolism from -omics data. 

Context-specific FBA on genome-scale metabolic networks (GEMs) has been used for decades to understand how gene and protein expression is coordinated to regulate metabolism in humans and human cancers [15,16,17,18,19]. Within the systems biology community, metabolic modeling is a mature and robust field. International collaborative efforts have given rise to comprehensive mathematical models containing essentially every known metabolic reaction in organisms ranging from the simplest obligate parasites to complex eukaryotes, as well as a range of disease states, including many cancers. However, the applications of metabolic modeling in cancer have been largely limited to “consensus” models, comprising mathematical descriptions of an average or representative tumor. While such models are valuable in elucidating potential new drug targets and understanding cancer metabolism broadly, they offer little insight into patient-to-patient variability in cancer metabolism. Recently, a method for integrating gene expression with GEMs was developed called Genome-scale metabolic modeling with Enzymatic Constraints using Kinetic and Omics (or GECKO) [20,21]. Briefly, GECKO uses gene expression values of metabolic enzymes multiplied by enzyme metabolic turnover numbers (k_cat_s) downloaded from the BRENDA database as constraints on a pre-built human metabolic reconstruction [22]. The subsequent enzyme-constrained metabolic model is then solved using FBA, maximizing cell growth (or biomass production) subject to these enzymatic constraints. 

Originally, GECKO was designed to be used with proteomic data to constrain metabolic behavior, under the assumption that protein expression would be more closely related to metabolism than transcript expression. However, in this study, we used RNA-seq data instead of protein data because of the large quantity and high quality of available RNA-seq data paired with outcomes in the TCGA database. This provides both quantitative depth (i.e., absolute quantitation at the genome scale) and breadth (multiple cancer types) to explore the relationship between metabolism and patient outcomes. Ultimately, both protein measurement and RNA measurement are surrogate markers for protein activity, which would be the most relevant measurement for metabolic modeling. While RNA expression is not a perfect representation of protein activity, we hypothesize that there is enough information in genome-scale RNA expression to extract meaningful results from a metabolic modeling approach. 

Here, we expanded the original GECKO modeling approach by integrating both internal constraints (from an in-house modified version of the GECKO algorithm) and external constraints from serum metabolite concentrations downloaded from the Human Metabolome Database (HMDB) and models of blood flow through the human body [23]. We employed context-specific FBA to characterize metabolism across 10,915 patients from 34 cancer types from the TCGA consortium and MMRF-COMMPASS databases, ranging from 1041 patients in breast cancer to 85 patients in mesothelioma. We then constructed metabolic profiles across this diverse range of individuals and cancer types. This approach enables us to assess the wealth of variability present across cancers, as well as within specific cancer types. We then interrogated these metabolic profiles to identify prognostic features across cancer types, with a focus on metabolic specific growth rate. Finally, we used similarities in prognostic profiles to infer therapies that could be expanded across cancer indications.

## 2. Materials and Methods

Key resources used in this study are highlighted in Table 1. 

### 2.1. RNA-Seq Data Acquisition

RNA-seq data were downloaded as transcripts per million mapped reads (TPM) from TCGA and the MMRF-COMMPASS study [24,25,26]. No additional processing of the RNA-seq data or batch correction was performed.

### 2.2. GEM Construction and Validation

GEMs for MM were constructed using an in-house modified version of HMR2 [16]. We first constrained metabolite input fluxes based on concentrations of serum metabolites downloaded from the Human Metabolome Database (HMDB), mapped to HMR reaction IDs (see Supplemental Table S1), and scaled by a model of blood flow through the human body. We further constrained these models using an in-house modified version of the GECKO formalism that we have optimized for stability in response to perturbations in metabolite availability. The GECKO formalism has been extensively described elsewhere [20,21]. Briefly, GECKO uses gene expression values of metabolic enzymes multiplied by enzyme metabolic turnover numbers (k_cat_s) downloaded from the BRENDA database as constraints on a pre-built human metabolic reconstruction [22]. Assuming non-limiting reactants, the maximum reaction rate for a reaction (r) is therefore dependent on the expression level of an enzyme (E) and its turnover number, as in Equation (1), below:

r ≤ E × k_cat_ (1)

The subsequent enzyme-constrained metabolic model is then solved using FBA, maximizing cell growth (or biomass production) subject to these enzymatic constraints. Taken together, our algorithm expands on the GECKO methodology by combining gene expression data and catalytic turnover numbers from the BRENDA database with physiologically determined external constraints on metabolite fluxes (see also Table S1).

For each cancer type, a minimum amount of maintenance ATP demand was imposed as a constraint in the model. The value used for this ATP demand was previously optimized in-house for our metabolic modeling approach (See Section 2.3, below). For each cancer type, the median expression (in TPM) of each gene was then calculated to create a “median expression profile” for each cancer type. This median expression profile was then simulated using FBA and the methods described above to maximize cell growth. If no growth was possible (model was infeasible), the constraint for maintenance ATP demand was decreased by half, and the model was simulated again. In all cases, cancers with infeasible solutions at high ATP maintenance values had feasible FBA solutions at the lower ATP maintenance demand constraint. 

All metabolic model simulations were implemented in Python using the COBRA toolbox [27].

### 2.3. ATP Flux Constraints

The ATP demand in these models was initially set at 0.05 mmol/gDwt/h, which is based on a 2000 kcal daily diet giving rise to an estimated maximum possible ATP flux of 0.40169 mmol/gDwt/h. The value of 0.40169 mmol/gDwt/h was then heuristically reduced to 0.05 mmol/gDwt/h to account for digestive, metabolic, and nutrient delivery inefficiencies and that not all nutrients are used for maintenance ATP.

### 2.4. Statistical Analyses

Principal component analysis and varimax rotation were performed in R using the base R functionality [28,29].

### 2.5. Survival Analysis

Survival analysis was performed using Kaplan–Meier curves for univariate analysis and Cox proportional hazard regression for multivariate analysis using the survminor package in R [30,31]. All simulated growth rates or subsystem fluxes were binarized using the median value per cancer type to determine “high” vs. “low” for survival analysis, except where specified in the manuscript as quantile separation. *p*-values were calculated using the built-in log-likelihood test. A *p*-value of ≤0.05 was considered statistically significant, while a *p*-value ≤ 0.10 was considered moderately associated with survival. 

## 3. Results

### 3.1. Simulating Cell Metabolism and Growth across 34 Cancer Types

**Figure 1:**
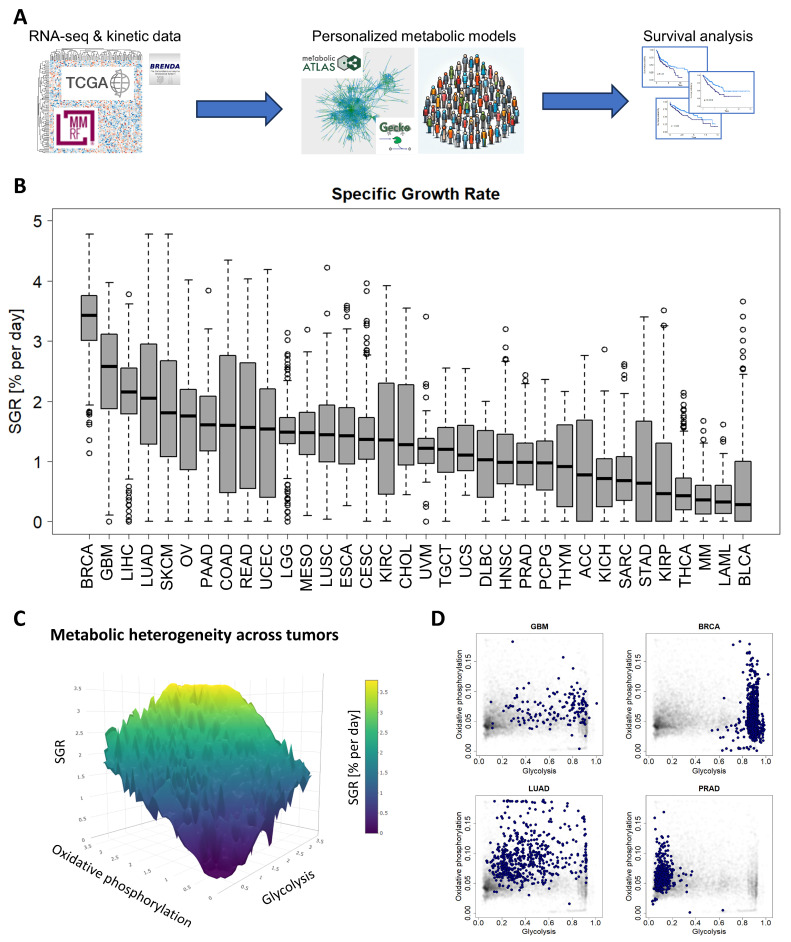
A standardized pipeline for simulating metabolism identified metabolic commonalities and differences across 10,915 patients from 34 cancer types. (**A**) Study overview. RNA-seq and reaction kinetic data were downloaded from the TCGA, MMRF-COMMPASS, and BRENDA databases. Genome-scale metabolic modeling using the GECKO algorithm was performed to characterize individual metabolic profiles for each patient from RNA expression profiles. Finally, survival analyses were performed to associate metabolic activities with survival within cancer types. (**B**) Metabolic specific growth rates (SGRs) across 34 cancer types showed a significant overlap in growth rates across cancer types. (**C**) Metabolic heterogeneity was governed by a combination of SGR, glycolysis pathway activity, and oxidative phosphorylation pathway activity. Highest SGR tumors have high activity of both glycolysis and oxidative phosphorylation pathways. (**D**) Specific cancer types tend to occupy characteristic regions of the glycolysis/oxidative phosphorylation space. GBM and BRCA tend to have high glycolysis, and low oxidative phosphorylation. LUAD has a range of glycolysis and oxidative phosphorylation. PRAD tends to have higher oxidative phosphorylation and low glycolysis.

We first performed FBA using an in-house modified version of the GECKO algorithm on an in-house curated version of the Human Metabolic Reconstruction 2.0 (HMR2) network [16] to optimize cell growth across 10,915 patients from 34 cancer types from TCGA consortium and MMRF-COMMPASS study (Figure 1A). We began by calculating the median expression of each gene within a cancer type (for example, glioblastoma or GBM) and constructing a “median” profile for that cancer. We then tested whether this median patient could grow with an ATP maintenance flux constraint (i.e., maintenance ATP demand) (See Section 2.3).

We found that the following cancer types had a reduced capacity for ATP maintenance: cervical squamous cell carcinoma and endocervical adenocarcinoma (CESC), cholangiocarcinoma (CHOL), esophageal carcinoma (ESCA), head and neck squamous cell carcinoma (HNSC), acute myeloid leukemia (LAML), lung squamous cell carcinoma (LUSC), mesothelioma (MESO), multiple myeloma (MM), pancreatic adenocarcinoma (PAAD), prostate adenocarcinoma (PRAD), sarcoma (SARC), testicular germ cell tumors (TGCT), thyroid carcinoma (THCA), and uterine carcinosarcoma (UCS). We expected some of these to be slow-growing, such as PRAD [32], which aligns with our finding that these cancers appear metabolically limited. Others, like LAML and LUSC, tend to be aggressive cancers [33,34,35], so it was surprising to find that these tumors had reduced capacity for ATP production. 

We next simulated metabolism in each of 10,915 individual patients across 34 cancer types and compared variability of metabolic specific growth rate (SGR) across cancer types, as a standardized metric for assessing proliferation (Figure 1B) [36]. We found that there was a large overlap in the distributions of individual patient SGRs across cancer types. For example, we observed that most GBM tumors grew quickly and most MM cancers grew slowly (Figure 1B). Nevertheless, the GBM and MM SGR distributions overlap to a significant extent. 

We next tested which metabolic subsystems were contributing to the heterogeneity seen across patients. Using a combination of principal component analysis (PCA) and varimax rotation, we found that glycolysis is the dominant metabolic feature contributing to cross-cancer variability and therefore changes in cancer growth rate (with ~94% of variability directly attributable to glycolysis). The remaining variability could largely be attributed to 2 metabolic pathways: oxidative phosphorylation (~2% of variability) and the pentose phosphate pathway (~1% of variability). Relating glycolysis and oxidative phosphorylation to cancer growth rate (~96% of intratumoral metabolic variability) suggests a “fitness landscape” of cell states that cancers traverse (Figure 1C). Cancers can grow by enhancing glycolysis, as in GBM or breast cancer (BRCA); by enhancing oxidative phosphorylation, as in PRAD; or by a combination of both, as in lung adenocarcinoma (LUAD) (Figure 1D and Supplemental Figure S1). This landscape gives insight into the adaptability of cancer subtypes and their flexibility to optimize cellular metabolism depending on the specific tumor microenvironment.

### 3.2. Metabolic SGR Is Prognostic across Cancer Types

**Figure 2:**
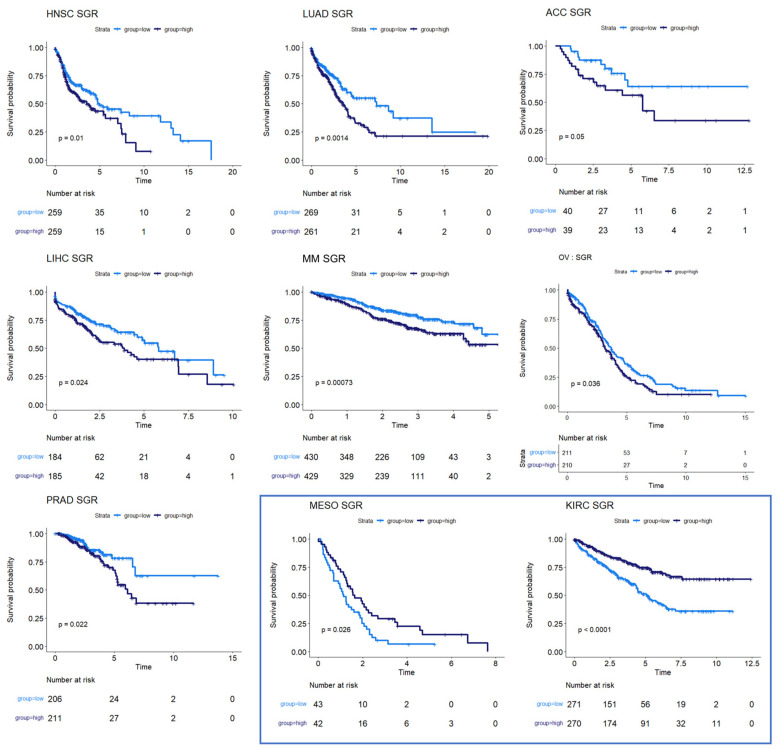
Survival analysis for cancers where SGR was prognostic of survival. KM plots show that high SGR was associated with worse overall survival prognostic in HNSC, LUAD, adrenocortical carcinoma (ACC), LIHC, MM, and OV patients. In PRAD, SGR was prognostic of RFS, with higher SGR patients having worse prognoses. (Boxed insert) In both MESO and KIRC, high SGR was associated with better prognoses.

**Figure 3:**
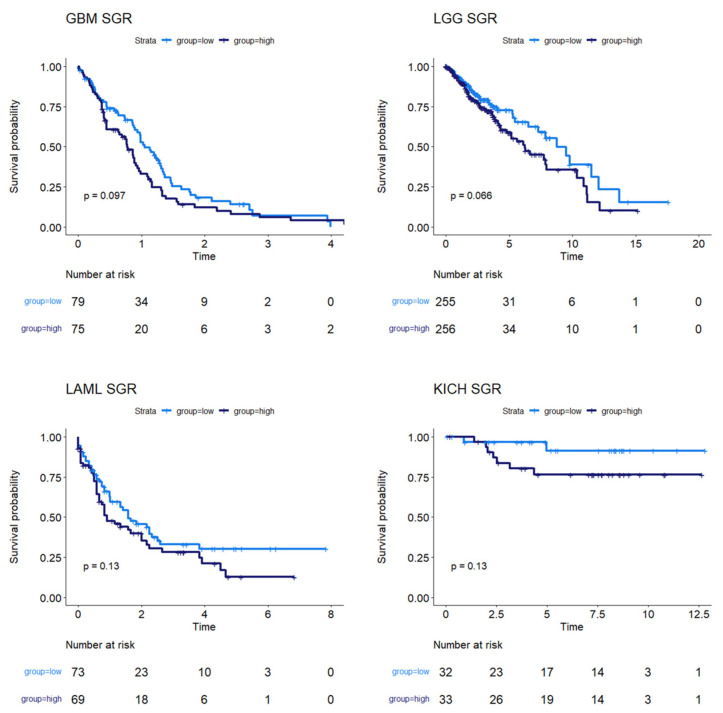
Survival analysis for cancers where SGR was moderately prognostic of survival. KM plots show that, in GBM, LGG, LAML, and KICH, SGR was moderately prognostic of overall survival (*p*-value ≤ 0.10), with higher SGR patients having worse prognoses.

We next used survival data collected by TCGA and the MMRF-COMMPASS study to test whether SGR was prognostic across cancers. For each cancer type, we binarized SGR into high vs. low based on the median from that cancer type. For PRAD, we used regression-free survival, RFS, as an endpoint instead of OS because of the few OS events in this data set. We found that SGR was strongly prognostic in 9 cancer types (*p*-value ≤ 0.05) (Figure 2) and moderately prognostic in another 4 (*p*-value ≤ 0.10) (Figure 3). In nearly all these cases, high SGR was associated with worse overall survival. In MESO and Kidney renal clear cell carcinoma (KIRC), however, high SGR was associated with better survival, possibly due to high responsiveness to growth-targeting chemotherapies (Figure 2, boxed KM plots).

### 3.3. Subtype Analysis in Breast Cancer

**Figure 4:**
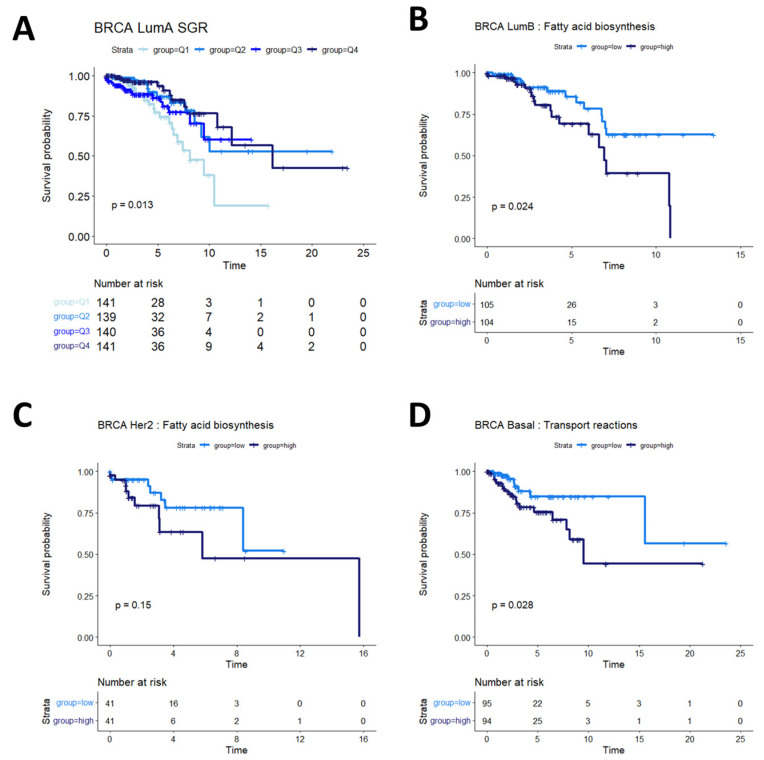
Survival analysis in breast cancer subtypes. (**A**) In LumA BRCA patients, SGR was prognostic of overall survival when split by quantiles, with high-SGR patients having worse prognosis. (**B**) In LumB BRCA patients, fatty acid biosynthesis was prognostic of overall survival, with higher activity associated with worse prognosis. (**C**) In Her2 BRCA patients, fatty acid synthesis appeared moderately associated with overall prognosis (*p* = 0.15); more patients would be required to validate this association in this subtype. (**D**) In basal BRCA patients, activity of transport reactions was associated with overall survival, with higher transport activity associated with worse prognosis.

Breast cancers are often sub-stratified both clinically and in research settings. One method in use for breast cancer stratification is the PAM50 classification, which is an expression panel of 50 genes that identifies the intrinsic molecular subtype of breast cancer tumors that has been shown to be prognostic of overall survival [37]. Based on this subtyping, we investigated survival in Luminal A (LumA), Luminal B (LumB), HER2-enriched (Her2), and basal-like (Basal) breast cancers from the TCGA BRCA cohort. We found that SGR was prognostic in LumA BRCA, with faster-growing tumors having a better prognosis than slow-growing tumors (Figure 4A). SGR was not prognostic in the other subtypes in a univariate analysis, split by median SGR or by quantiles. Instead, fatty acid biosynthesis appeared to be prognostic in LumB BRCA tumors (Figure 4B), and potentially prognostic in Her2 BRCA tumors (Figure 4C). The relatively small number of samples in the Her2 cohort makes identifying statistical discernability difficult in this population. In basal BRCA tumors, the metabolic flux of transport reactions between cellular compartments was significantly associated with overall survival (Figure 4D). The main drivers of this phenomenon were transport of extracellular metabolites into the cytosol and transport across the mitochondrial membrane, suggesting that basal breast cancers pull nutrients from diverse sources, but the pinch point for effective therapies may reside in mitochondrial transport. 

### 3.4. Folate Metabolism Is Prognostic across Several Cancers

**Figure 5:**
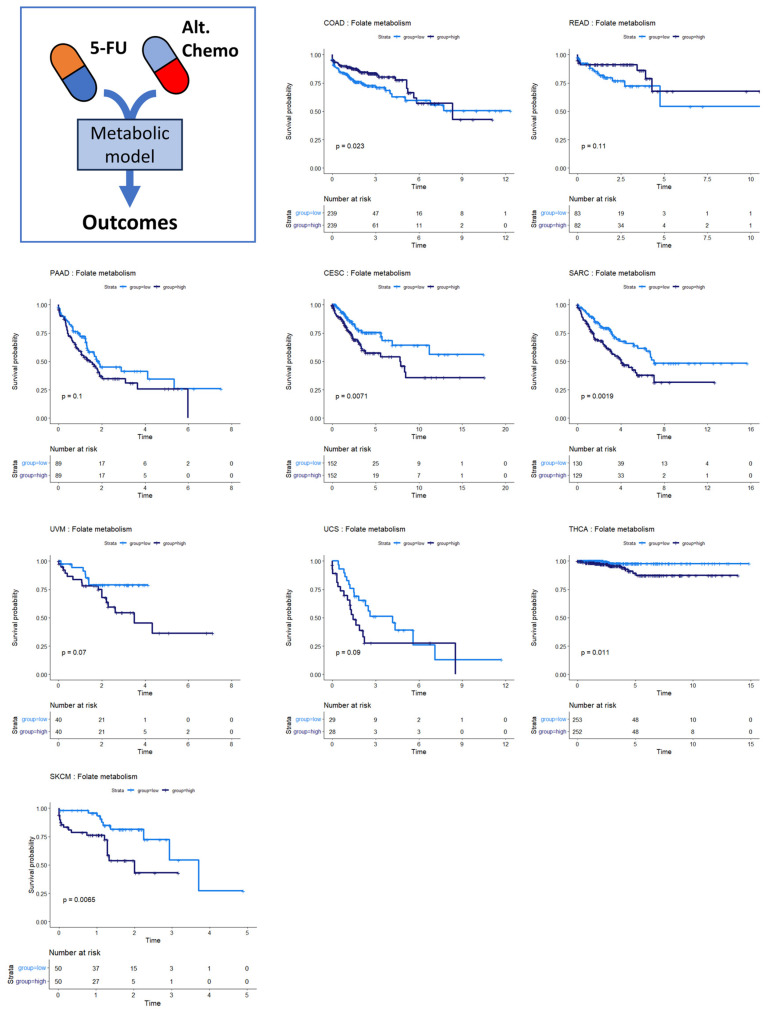
Survival analysis for cancers where folate metabolism was prognostic of survival. Across multiple cancer types, folate metabolism was associated with overall survival. In COAD/READ, higher folate metabolism activity was associated with better prognosis. In PAAD, CESC, SARC, UVM, UCS, THCA, and SKCM, high folate metabolism activity was associated with worse prognosis.

Several of the cancer types investigated had no discernible relationship between SGR and overall survival. Overall survival in two of these cancer types, colon and rectal cancer, was instead associated with differential activity of folate metabolism. Patients whose tumors had high activity of folate metabolism had a higher survival probability than those with low activity of folate metabolism (Figure 5, top row). This is perhaps because colorectal cancers are often treated with 5-FU based chemotherapies targeting folate metabolism, including FOLFOX, FOLFRI, and FOLFIRINOX [38].

We also found that folate metabolism appeared somewhat prognostic in pancreatic adenocarcinoma (PAAD) (Figure 5) (*p* = 0.10). For PAAD, however, the survival is reversed: high folate metabolism was associated with poor survival, while low folate metabolism was associated with better survival. While 5-FU was incorporated into several therapies during the time the TCGA PAAD cohort was accruing—most notably 5-FU combined with radiation—gemcitabine monotherapy was a dominant first-line therapy until 2011. Thereafter, clinical trials showed that FOLFIRINOX enhanced survival compared to gemcitabine for metastatic pancreatic cancer patients. Together, these findings suggest that the survival characteristics in the PAAD KM plot were primarily driven by response to gemcitabine therapy rather than antifolate-based therapies [39,40]. Several other cancer types show the same pattern of survival as PAAD: cervical squamous cell carcinoma and endocervical adenocarcinoma (CESC), sarcoma (SARC), uveal melanoma (UVM), uterine carcinosarcoma (UCS), thyroid carcinoma (THCA), and skin cutaneous melanoma (SKCM). This pattern suggests that 5-FU-based therapies like FOLFIRINOX could be beneficial in these patient populations. Indeed, there is evidence that suggests this is the case [41,42,43,44,45].

### 3.5. Other Metabolic Trends in Survival Data

**Figure 6:**
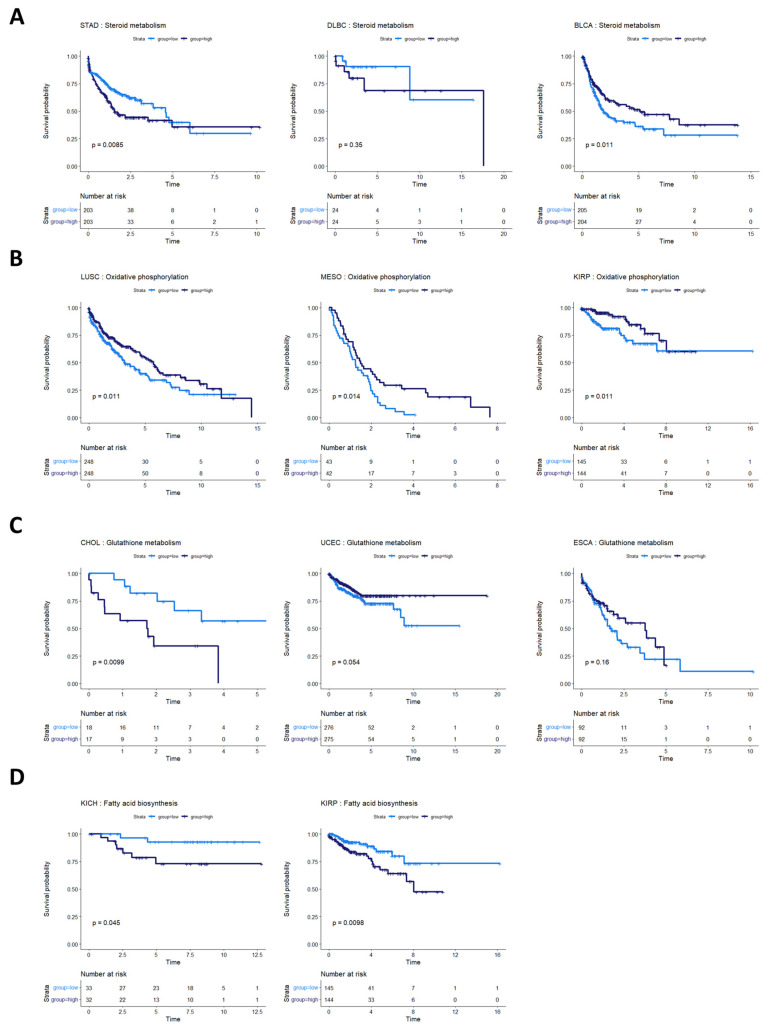
Survival analysis for cancers with other metabolic pathway activities prognostic of survival. (**A**) Survival analysis for cancers where steroid metabolism was prognostic of survival. In STAD, BLBC, and BLCA, steroid metabolism pathway activity appears to be associated with overall survival. In DLBC, more patients are required to validate this effect. (**B**) Cancers where oxidative phosphorylation was prognostic of overall survival. High oxidative phosphorylation was associated with better prognosis in LUSC, MESO, and KIRP. (**C**) Glutathione metabolism pathway activity appeared prognostic for CHOL, UCEC, and ESCA. (**D**) Fatty acid biosynthesis activity appeared prognostic in two kidney cancers, KICH and KIRP.

Steroid metabolism was prognostic of overall survival in three cancer types: stomach adenocarcinoma (STAD), bladder urothelial carcinoma (BLCA), and lymphoid neoplasm diffuse large B-cell lymphoma (DLBC) (although not statistically discernable at a *p*-value of 0.05 in DLBC, potentially because of the small sample size and low number of survival events) (Figure 6A). These survival profiles suggest that steroid-based therapies could be targeted to at least subsets of patients in these cancer types. Indeed, standard treatment for DLBC cancers already involves steroid-based regimens [46,47]. Bladder cancer patients, in particular, might benefit from targeted application of steroid therapies because studies have shown both an inhibitory and a promotional role of steroids on bladder cancer growth [48,49]. The metabolic heterogeneity identified in our study could help explain these apparently contradictory findings in STAD. The consideration of whether to use steroids in stomach cancer treatment could potentially balance the likelihood of benefit from a metabolic model with findings indicating that pre-operative steroid use could complicate gastric cancer surgeries [50].

The activities of several other metabolic pathways were prognostic in various cancer types. Oxidative phosphorylation was prognostic in LUSC, MESO, and kidney renal papillary cell carcinoma (KIRP) (Figure 6B). Glutathione metabolism was prognostic in CHOL, UCEC, and esophageal carcinoma (ESCA) (although the latter two did not reach statistical discernability at a *p*-value of 0.05) (Figure 6C). Fatty acid biosynthesis was prognostic in two kidney cancer types, KIRP and KIRC (Figure 6D). 

Three cancer types showed no association between metabolic features and overall survival. These cancers were pheochromocytoma and paraganglioma (PCPG), testicular germ cell tumors (TGCT), and thymoma (THYM). The small sample sizes of tumors from these collections (179, 150, and 120, respectively) and the low number of survival events in the clinical data (6, 4, and 9, respectively) made connecting metabolic features to survival events particularly challenging. Table 2 summarizes the cancers investigated and the metabolic subsystem highlighted as associated with prognosis for each cancer type in this study.

## 4. Discussion

This study, to the best of our knowledge, represents the first large-scale evaluation of survival outcomes across multiple cancer types based on patient-specific genome-scale metabolic modeling. Using TCGA and the MMRF-COMMPASS RNA-seq databases, we implemented a standardized pipeline to assess metabolic function at a genomic scale in 34 cancer types. Our pipeline used “harmonized” TPM data from TCGA that was generated by (1) aligning reads to the Hg38 draft of the human genome using STAR alignment, and (2) using standardized parameters to extract unstranded TPM values for all reads. We used these TPM values with our in-house modified versions of GECKO and HMR2 and pre-determined parameter values to simulate metabolism across these diverse cancer types. Using a standardized pipeline as described here is beneficial because it allows for the analysis of new patient data without the need for additional normalization or batch correction techniques, such as ComBat or RVUSeq [51,52]. We demonstrated that this standardized analysis pipeline robustly associated metabolic features and overall survival. The flexibility of this method has applicability to researchers by enabling interrogation of large datasets and applicability to clinicians by enabling analysis of an individual patient’s cancer metabolic profile in the context of TCGA patient samples.

Several limitations to this study exist. Most notably, the survival analyses performed in this work are univariate and did not consider other possible confounding variables or comorbidities. It would not be surprising to find an association between tumor size (or stage) and metabolic SGR for newly diagnosed tumors. Indeed, tumors caught later are likely to be both larger and more aggressive. Additionally, biological and lifestyle features like sex, BMI, age, and smoking likely further influence survival within certain cancer types. Furthermore, cancer itself is a metabolic disturbance to a patient’s normal metabolic functionality, disrupting glucose bioavailability, serum lactate, and systemic metabolic pathways. Therefore, a careful metabolic characterization of individual patients will likely be critical for applying these types of models to understand patient tumors fully. 

As an additional limitation, we note that each transcriptome represented within the TCGA data set represents bulk RNA-seq from a different patient’s tumor tissue. As such, it includes cancer cells as well as other cell types, including infiltrating lymphocytes, fibroblasts, etc. Thus, each patient’s model represents a composite profile of the metabolism within the tumor tissue, not the cancer by itself. While we expect that the cancer transcriptome is the bulk of the measurement, de-convoluting tumor, immune, and tissue metabolism remains an ongoing area of research. 

Nevertheless, analyzing patient-specific metabolic profiles along with survival data in a univariate analysis does provide unique insights into the association between metabolism and prognosis. We found 9 cancer types where the metabolic SGR was prognostic of overall survival (*p* ≤ 0.05) and another 4 where it may be prognostic in larger data sets (*p* ≤ 0.10). It is also possible that sub-stratifying these cancer types into molecular phenotypes (as was done with breast cancer) could lead to the identification of new targeted therapies, which could be especially beneficial for fast-growing subtypes. 

In most of the cancer types where SGR was prognostic of survival, high SGR was associated with worse survival. This finding suggests that metabolic SGR can act as a surrogate for tumor aggressiveness, with more aggressive tumors having a worse prognosis independent of the therapy given. However, in two cancer types, MESO and KIRC, higher metabolic SGR was associated with better overall survival. We believe that this high growth rate/high survival phenotype may be related to the type of treatment given for these tumors or drug delivery and accumulation differences from other tumors; however, more research will be required to identify the biological mechanisms at work. 

Additionally, we found that certain groups of cancers appear to have similar prognostic landscapes, with folate metabolism, steroid metabolism, oxidative phosphorylation, glutathione metabolism, and fatty acid biosynthesis all prognostic in subsets of cancer types. The amount of flux through a subsystem may be associated with the dependency of that tumor on that pathway (e.g., cancers with high folate metabolism may be addicted to folate). Targeting these metabolic pathways in these cancer types is therefore a promising avenue for drug design or repurposing. It is also possible that cancers sharing similar metabolic pathways stratifying survival could be susceptible to similar therapies, suggesting the idea that therapies designed for one cancer type within a prognostic group could be beneficial for patients with other cancer types within the same grouping.

While this work has focused on associations between metabolic subsystems and survival, we understand that many clinicians and researchers are interested in associating single genes with overall survival as prognostic biomarkers or as novel drug targets. One of the key strengths of the modeling approach presented in this study is that we used composite pathway scores instead of using expression of single genes to associate metabolism and outcome data. This scoring allows us to view the pathway “as a whole”. While some pathways have enzymatic pinch points where the expression of one enzyme governs overall pathway flux across multiple patients, this is not always the case. Further analysis of the data generated in this study will allow for identification of single enzymes within prognostic subsystems that are associated with survival. In addition, gene essentiality computational experiments are an additional way to use the patient-specific models generated here to identify singly lethal and synthetically lethal metabolic knockouts for novel target identification. Furthermore, such knockout experiments in patient-specific models naturally generate estimates of population efficacy of inhibiting each of the proposed targets. 

## 5. Conclusions

This work takes the first steps towards using metabolic modeling to understand cancer biology with patient specificity. We anticipate that modeling approaches like the ones presented in this work are poised to begin working their way into the clinic. Armed with an individual patient’s tumor metabolic profile, doctors can begin to ask different questions about personalizing patient care. For example, “How does the growth rate of this tumor compare to those from other patients with the same cancer type?” “What metabolic pathways deviate from expected values based on a large cohort of patients?” and “Are those pathways druggable?” Ultimately, we believe that metabolic modeling will be one piece of a comprehensive modeling platform for patient tumors that will allow doctors to personalize care considering multiple types of data, from imaging to sequencing, within a unified framework to help personalize care for cancer patients.

## Figures and Tables

**Table 1:** Key resources table.

Reagent or Resource	Source	Identifier
Deposited data		
TCGA RNA-seq data	NIH/NCI	https://portal.gdc.cancer.gov/ (accessed on 21 December 2023)
MMRF-COMMPASS study RNA-seq data	Multiple Myeloma Research Foundation	https://portal.gdc.cancer.gov/ (accessed on 21 December 2023)
Human Metabolome Database (HMBD)	Canadian Institutes of Health Research, Canada Foundation for Innovation, and The Metabolomics Innovation Centre (TMIC)	https://hmdb.ca/ (accessed on 2 March 2020)
BRENDA database	Leibniz Institute DSMZ	https://www.brenda-enzymes.org/ (accessed on 20 February 2022)
Software and algorithms		
HMR 2.0	The Metabolic Atlas project	https://metabolicatlas.org/gems/repository (accessed on 20 February 2022)
R version 4.3.1	The R Foundation	https://cran.r-project.org/ (accessed on 16 June 2023)
RStudio version 2023.06.0+421 “Mountain Hydrangea” Release (583b465ecc45e60ee9de085148cd2f9741cc5214, 2023-06-05) for Windows	Posit Software, PBC	https://posit.co/download/rstudio-desktop/ (accessed on 16 June 2023)
Python version 3.8.18	Python Software Foundation	https://www.python.org/ (accessed on 10 November 2023)

**Table 2:** Summary of cancer types investigated and metabolism subsystems associated with survival.

Cancer Type ID	Cancer Type Name	Metabolic Subsystem	High Flux vs. Prognosis	*p*-Value
ACC	Adrenocortical carcinoma	SGR	Poor	0.05
BLCA	Bladder urothelial carcinoma	Steroid metabolism	Poor	0.011
BRCA basal	Breast invasive carcinoma	Transport reactions	Poor	0.028
BRCA Her2	Breast invasive carcinoma	Fatty acid biosynthesis	Poor	0.15
BRCA LumA	Breast invasive carcinoma	SGR	Good	0.013
BRCA LumB	Breast invasive carcinoma	Fatty acid biosynthesis	Poor	0.024
CESC	Cervical squamous cell carcinoma and endocervical adenocarcinoma	Folate metabolism	Poor	0.0071
CHOL	Cholangiocarcinoma	Glutathione metabolism	Poor	0.0099
COAD	Colon adenocarcinoma	Folate metabolism	Good	0.023
DLBC	Lymphoid neoplasm diffuse large B-cell lymphoma	Steroid metabolism	Poor	0.35
ESCA	Esophageal carcinoma	Glutathione metabolism	Good	0.16
GBM	Glioblastoma multiforme	SGR	Poor	0.097
HNSC	Head and neck squamous cell carcinoma	SGR	Poor	0.01
KICH	Kidney chromophobe	SGR	Poor	0.13
KICH	Kidney chromophobe	Fatty acid biosynthesis	Poor	0.045
KIRC	Kidney renal clear cell carcinoma	SGR	Good	<0.0001
KIRP	Kidney renal papillary cell carcinoma	Oxidative phosphorylation	Good	0.011
KIRP	Kidney renal papillary cell carcinoma	Fatty acid biosynthesis	Poor	0.0098
LAML	Acute myeloid leukemia	SGR	Poor	0.13
LGG	Brain lower-grade glioma	SGR	Poor	0.066
LIHC	Liver hepatocellular carcinoma	SGR	Poor	0.024
LUAD	Lung adenocarcinoma	SGR	Poor	0.0014
LUSC	Lung squamous cell carcinoma	Oxidative phosphorylation	Good	0.011
MESO	Mesothelioma	SGR	Good	0.026
MESO	Mesothelioma	Oxidative phosphorylation	Good	0.014
MM	Multiple myeloma	SGR	Poor	0.0007
OV	Ovarian serous cystadenocarcinoma	SGR	Poor	0.036
PAAD	Pancreatic adenocarcinoma	Folate metabolism	Poor	0.1
PCPG	Pheochromocytoma and paraganglioma	None	N/A	N/A
PRAD	Prostate adenocarcinoma	SGR	Poor	0.022
READ	Rectum adenocarcinoma	Folate metabolism	Good	0.11
SARC	Sarcoma	Folate metabolism	Poor	0.0019
SKCM	Skin cutaneous melanoma	Folate metabolism	Poor	0.0065
STAD	Stomach adenocarcinoma	Steroid metabolism	Poor	0.0085
TGCT	Testicular Germ Cell Tumors	None	N/A	N/A
THCA	Thyroid carcinoma	Folate metabolism	Poor	0.011
THYM	Thymoma	None	N/A	N/A
UCEC	Uterine corpus endometrial carcinoma	Glutathione metabolism	Good	0.054
UCS	Uterine carcinosarcoma	Folate metabolism	Poor	0.09
UVM	Uveal melanoma	Folate metabolism	Poor	0.07

## Data Availability

The original contributions presented in the study are included in the article/Supplementary Materials, further inquiries can be directed to the corresponding author.

## Supplementary Materials

The following supporting information can be downloaded at: https://www.mdpi.com/article/10.3390/cancers16132302/s1, Table S1: Metabolite concentrations mapped to HMR2 reactions; Figure S1: Glycolysis vs. Oxidative Phosphorylation landscapes across 34 cancer types.
