# Supplementary figures and images for: Pan-Cancer, Genome-Scale Metabolic Network Analysis of over 10,000 Patients Elucidates Relationship between Metabolism and Survival
Source: Cancers (Basel). 2024 Jun 22;16(13):2302. doi: 10.3390/cancers16132302 (PMC11240338; doi:10.3390/cancers16132302)

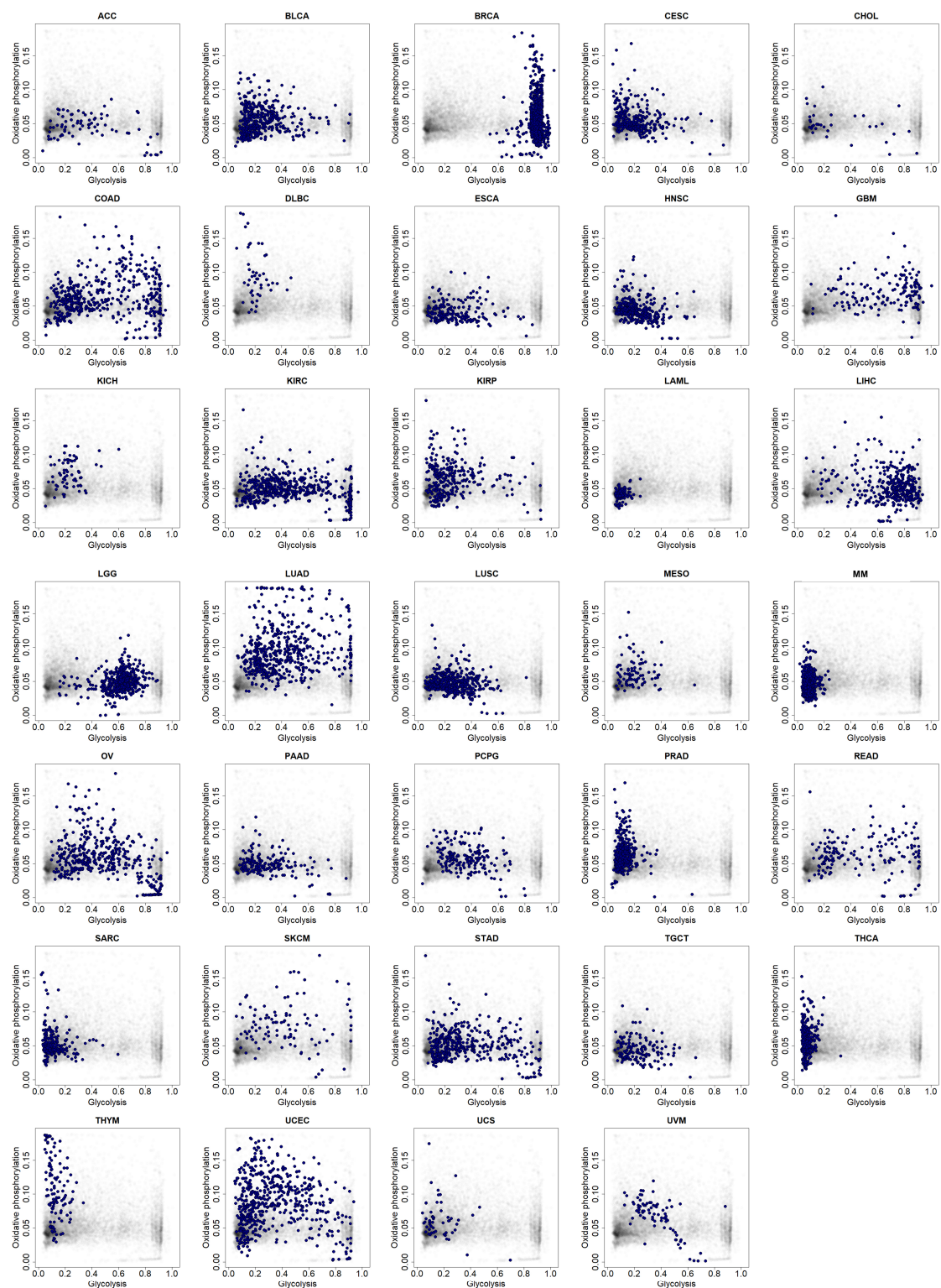

**Figure S1.** Glycolysis vs. Oxidative Phosphorylation landscapes across 34 cancer types.

Supplement: Supplementary file 1 [file cancers-16-02302-s001.zip › Figure S1.pdf]
